# Introduction of a breast apparent diffusion coefficient category system (ADC-B) derived from a large multicenter MRI database

**DOI:** 10.1007/s00330-023-09675-0

**Published:** 2023-05-11

**Authors:** Hubert Bickel, Paola Clauser, Katja Pinker, Thomas Helbich, Iva Biondic, Boris Brkljacic, Matthias Dietzel, Gordana Ivanac, Barbara Krug, Marco Moschetta, Victor Neuhaus, Klaus Preidler, Pascal Baltzer

**Affiliations:** 1 Dpt. of Biomedical Imaging and Image Guided Therapy, Medical University Vienna, Waehringer Guertel 18-20, 1090 Vienna, Austria; 2 Diagnosezentrum Meidling, Meidlinger Hauptstr. 7 – 9, 1120 Vienna, Austria; 3 Evelyn H. Lauder Breast Center, Memorial Sloan Kettering Cancer Center, 300 East 66th Street, New York, NY 10065 USA; 4 Dpt. of Diagnostic and Interventional Radiology, University Hospital Dubrava, University of Zagreb School of Medicine, Avenija Gojka Šuška 6, 10 000 Zagreb, Croatia; 5 Dpt. of Radiology, University Hospital Erlangen, Maximiliansplatz 3, 91054 Erlangen, Germany; 6 Dpt. of Diagnostic and Interventional Radiology, University Hospital Cologne, Kerpener Str. 62, 50937 Cologne, Germany; 7 Dpt. of Emergency and Organ Transplantation-Breast Care Unit, Aldo Moro University of Bari Medical School, Piazza Giulio Cesare 11, 70124 Bari, Italy

**Keywords:** Diffusion magnetic resonance imaging, Breast neoplasms, Classification

## Abstract

**Objectives:**

To develop an intuitive and generally applicable system for the reporting, assessment, and documentation of ADC to complement standard BI-RADS criteria.

**Methods:**

This was a multicentric, retrospective analysis of 11 independently conducted institutional review board–approved studies from seven institutions performed between 2007 and 2019. Breast Apparent Diffusion coefficient (ADC-B) categories comprised ADC-B0 (ADC non-diagnostic), ADC-B1 (no enhancing lesion), and ADC-B2-5. The latter was defined by plotting ADC versus cumulative malignancy rates. Statistics comprised ANOVA with post hoc testing and ROC analysis. *p* values ≤ 0.05 were considered statistically significant.

**Results:**

A total of 1625 patients (age: 55.9 years (± 13.8)) with 1736 pathologically verified breast lesions were included. The mean ADC (× 10^−3^ mm^2^/s) differed significantly between benign (1.45, SD .40) and malignant lesions (.95, SD .39), and between invasive (.92, SD .22) and in situ carcinomas (1.18, SD .30) (*p* < .001). The following ADC-B categories were identified: ADC-B0—ADC cannot be assessed; ADC-B1—no contrast-enhancing lesion; ADC-B2—ADC ≥ 1.9 (cumulative malignancy rate < 0.1%); ADC-B3—ADC 1.5 to < 1.9 (0.1–1.7%); ADC-B4—ADC 1.0 to < 1.5 (10–24.5%); and ADC-B5—ADC < 1.0 (> 24.5%). At the latter threshold, a positive predictive value of 95.8% (95% CI 0.94–0.97) for invasive versus non-invasive breast carcinomas was reached.

**Conclusions:**

The breast apparent diffusion coefficient system (ADC-B) provides a simple and widely applicable categorization scheme for assessment, documentation, and reporting of apparent diffusion coefficient values in contrast-enhancing breast lesions on MRI.

**Clinical relevance statement:**

The ADC-B system, based on diverse MRI examinations, is clinically relevant for stratifying breast cancer risk via apparent diffusion coefficient measurements, and complements BI-RADS for improved clinical decision-making and patient outcomes.

**Key Points:**

*• The breast apparent diffusion coefficient category system (ADC-B) is a simple tool for the assessment, documentation, and reporting of ADC values in contrast-enhancing breast lesions on MRI.*

*• The categories comprise ADC-B0 for non-diagnostic examinations, ADC-B1 for examinations without an enhancing lesion, and ADC-B2-5 for enhancing lesions with an increasing malignancy rate.*

*• The breast apparent diffusion coefficient category system may be used to complement BI-RADS in clinical decision-making.*

**Supplementary Information:**

The online version contains supplementary material available at 10.1007/s00330-023-09675-0.

## Introduction

Diffusion-weighted imaging (DWI) is a powerful tool to complement contrast-enhanced magnetic resonance (CE-MRI) imaging of the breast. It can be used as an imaging biomarker for the malignancy of breast tumors [1–3] and also for certain tumor features such as tumor invasiveness [4], or for treatment monitoring under neoadjuvant therapy [5].

DWI measures the random movement of water molecules by the application of diffusion gradients. This movement can be quantified by calculating the apparent diffusion coefficient (ADC). While many studies have shown the potential of DWI, its implementation into the breast clinical routine is still a work in progress: while the Breast Imaging Reporting and Data System (BI-RADS) has been established as a tool for the simple and comparable reporting of breast MRI [6], no categorization exists for DWI. Furthermore, a lack of technical standardization has led to discussion about the reproducibility and comparability of DWI measurements, hindering the broad application of this technique in clinical practice. In order to overcome these problems, an international working group has provided suggestions on technical standardization, and has suggested dividing ADC into categories [7] in order to make reporting more practicable. However, the suggested ADC categories are solely based on the ADC ranges of certain lesion subtypes, accumulated from a meta-analysis of previously published literature.

Thus, the aim of this retrospective study was to develop a simple and clinically applicable breast ADC (ADC-B) categorization system to complement MRI BI-RADS regarding the assessment, documentation, and reporting of ADC values in contrast-enhancing breast lesions on MRI, based on cumulative malignancy rates and ADC measurements from a large, multicentric breast MRI database.

## Materials and methods

### Study samples

Individual anonymized patient and lesion data from seven institutions in four countries were collected, pooled, and transferred into a multicenter database. The database included independent patient samples from eleven single-center studies, performed between 2007 and 2019. The data of the patients included in this analysis have in part been analyzed and published previously (1215 of 1625 patients; see Table 1) with different research questions. As opposed to these previous publications, in this study, the original data from the different studies were combined to develop an ADC categorization system.

**Table 1:** Numbers of included patients by center and previous publications of the patient data. All patients were female [23–31]

Center	No. of patients	Mean age (± SD)	No. of lesions	Benign (%)	Malignant (%)	Published in
Center 1	355	56.1 (14.8)	400	177 (44.3)	223 (55.8)	*4, 23–25*
Center 2	99	53.5 (13.7)	120	68 (56.7)	52 (43.3)	*23, 25, 26*
Center 3	144	56.4 (10.9)	144	10 (6.9)	134 (93.1)	*14, 25, 27*
Center 4	324	56.5 (13.6)	356	136 (38.2)	220 (61.8)	*25, 28–30*
Center 5	212	51.6 (12.6)	222	73 (32.9)	149 (67.1)	Unpublished data
Center 6	293	60.3 (14.2)	295	87 (29.5)	208 (70.5)	*25, 31*
Center 7	198	54.1 (12.7)	199	30 (15.1)	169 (84.9)	Unpublished data
*Overall*	1625	*55.9 (13.8)*	*1736*	*581 (33.5)*	*1155 (66.5)*	

### Patients

**Fig. 1:**
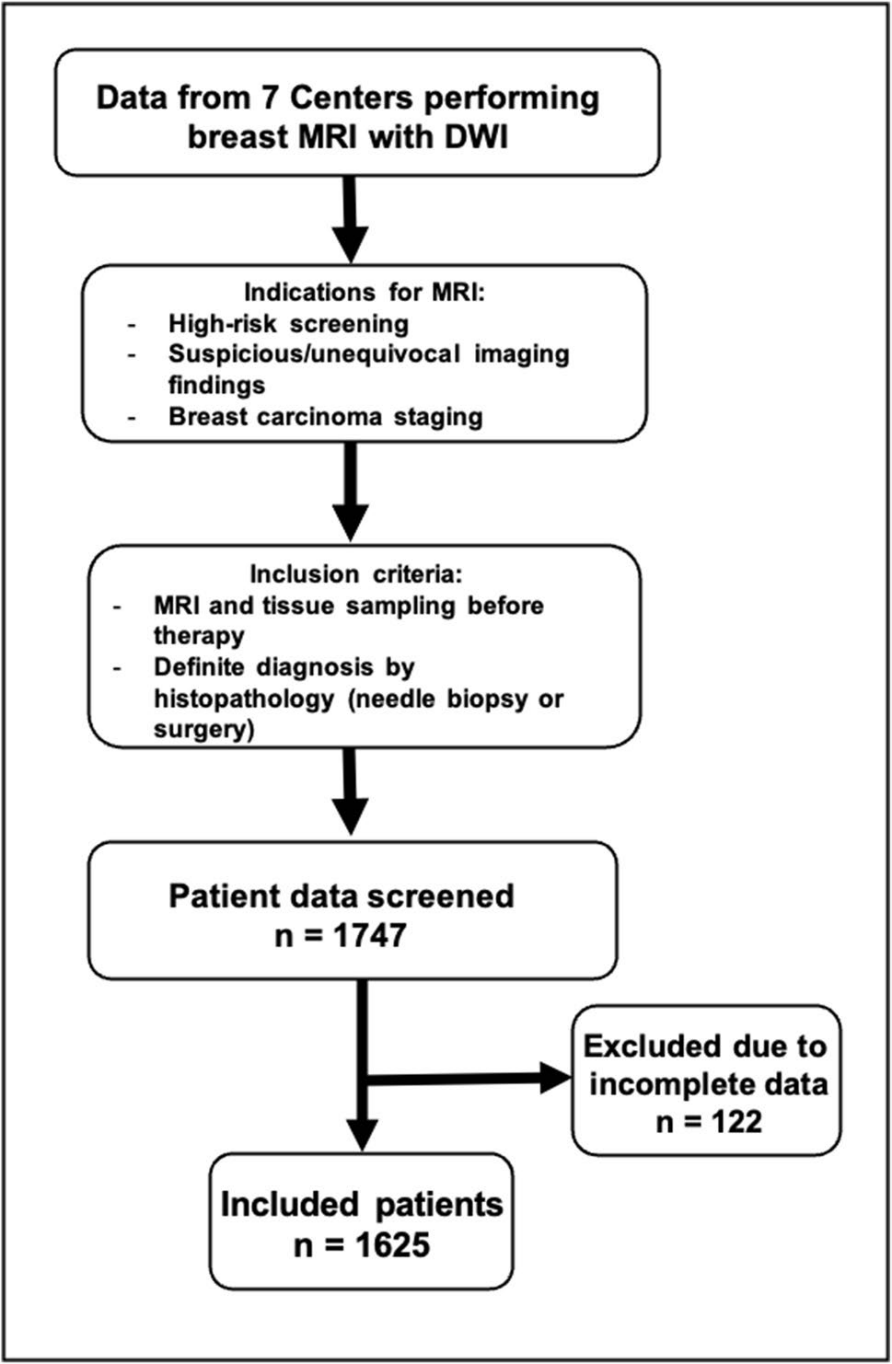
Flowchart depicting the included and excluded lesions. Abbreviations: MRI—magnetic resonance imaging; DWI—diffusion-weighted imaging

Indications for MRI, inclusion criteria, and exclusion criteria are displayed in Fig. 1. The numbers of included cases per center are displayed in Table 1. Each single-center study was approved by the local institutional review board. Because of the retrospective nature of the data analysis, the IRB waived the need for a signed informed consent. Data collection and aggregation was performed in a fully anonymized way and in line with international legislation.

The different histologic subtypes were summarized into the following categories for further analysis:

- Benign lesions (with a sub-category for high-risk lesions)
- Invasive breast carcinomas
- Invasive mucinous breast carcinomas
- Ductal carcinomas in situ (DCIS)
- Other malignancies (encompassing malignancies that do not belong to the aforementioned categories, such as metastases of extramammary tumors)

The category “high risk” was attributed to lesions of uncertain malignant potential, which were not malignant in the final histology after surgery or vacuum biopsy. The included high-risk lesions were atypical ductal hyperplasia, lobular carcinoma in situ/lobular neoplasia, atypical columnar cell hyperplasia, radial scar/complex sclerosing adenosis, flat epithelial atypia, papilloma/papillomatosis, and phyllodes tumor [8].

### MRI and ADC measurement techniques

All scans were performed on 1.5- or 3-T MRI scanners, using dedicated breast coils with the patients placed in prone position. All scans were performed using protocols that were standardized within each study sample following international guidelines, and included a T2-weighted sequence and native and CE T1-weighted sequences [6, 9]. All DWI scans were performed using echo planar imaging sequences and complied with the recommendations of the European Society of Breast Imaging (EUSOBI) [7]. ADC maps were calculated by each scanner’s integrated software using monoexponential fitting. Details on hardware, DWI scanning parameters, and image postprocessing used for the different patient collectives are displayed in Table 2.

**Table 2:** Hardware and sequence parameters as used for the different studies included in this retrospective analysis

Database	C1-P1	C1-P2	C2	C3	C4-P1	C4-P2	C4-P3	C5-P1	C5-P2	C6	C7
Scanner	Siemens Healthineers Trio Tim	Siemens Healthineers Magnetom Avanto	Siemens Healthineers Magnetom Espree	Siemens Healthineers Magnetom Avanto	Magnetom Sonata	Siemens Healthineers Magnetom Sonata	Siemens Healthineers Magnetom Avanto	Philips 1.5	Philips 3.0	Philips Achieva	Philips Ingenia
Field strength	3.0 T	1.5 T	1.5 T	1.5 T	1.5 T	1.5 T	1.5 T	1.5 T	3.0 T	1.5 T	1.5 T
Coil	InVivo 4-channel	InVivo 4-channel	Siemens Healthineers 4-channel	4-channel	Siemens Healthineers 4-channel	Siemens Healthineers 4-channel	Siemens Healthineers 4-channel	Philips 16-channel	Philips 16-channel	4-channel	7-channel
Plane	Axial	Axial	Axial	Axial	Axial	Axial	Axial	Axial	Axial	Axial	Axial
Diffusion sequence	SS-EPI	SS-EPI	SS-EPI	SS-EPI	SS-EPI	SS-EPI	SS-EPI	SS-EPI	SS-EPI	SS-EPI	SS-EPI
Fat suppression	IR	SPAIR	Water excitation	SPAIR	Spectral fat suppression	Spectral fat suppression	Spectral fat suppression	Spectral fat suppression	Spectral fat suppression	IR	Spectral fat suppression
TR/TE/TI (ms)	13700/83/220	6300/104/-	6300/117/-	7100/84/-	3500/80/-	3500/80/-	3500/73/-	83345/71/-	9897/72/-	6900/65/180	10900/92/-
Spatial resolution (mm)	1.8 × 1.8 × 3.5	2 × 2 × 4	1.6 × 1.6 × 3	2 × 2 × 4	1.8 × 1.8 × 6	1.8 × 1.8 × 6	1.8 × 1.8 × 6	0.3 × 0.3 × 3.0	0.3 × 0.3 × 3.0	3.1 × 3.1 × 3.0	2.5 × 2.5 × 3.0
*b* values (s/mm^2^)	50/850	50, 400, 800	0, 1000	0/1000	0, 750, 1000	0, 750, 1000	0, 750, 1000	0, 100, 300, 800	0, 100, 300, 800	0, 1000	0, 400, 800, 1200
Acquisition time (min:s)	3:19	2:00	2:50	2:29	2:48	2:38	2:30	6:09	5:23	2:04	2:45
ADC calculation	MF	MF	MF	MF	MF	MF	MF	MF	MF	MF	MF

*Cx-Px* center *X*-population *X*, *SS-EPI* single-shot echoplanar imaging, *IR* inversion recovery, *SPAIR* spectral adiabatic inversion recovery, *MF* monoexponential fit

All ADC measurements were performed using 2-dimensional regions of interest (ROIs) covering the darkest part of the lesion identified visually on the ADC map, while using the high-*b*-value DWI and CE T1-weighted images to avoid necrotic areas or low-signal areas caused by T2 blackout effects of fat suppression, according to recommendations of EUSOBI and a recent meta-analysis [7, 10, 11]. All measurements were performed independently by one or more radiologists blinded to histological outcome on clinical workstations. The radiologists had different levels of experience at breast MRI interpretation, ranging between 3 and 25 years (Supplemental Table 1).

### Breast ADC categories (ADC-B)

**Fig. 2:**
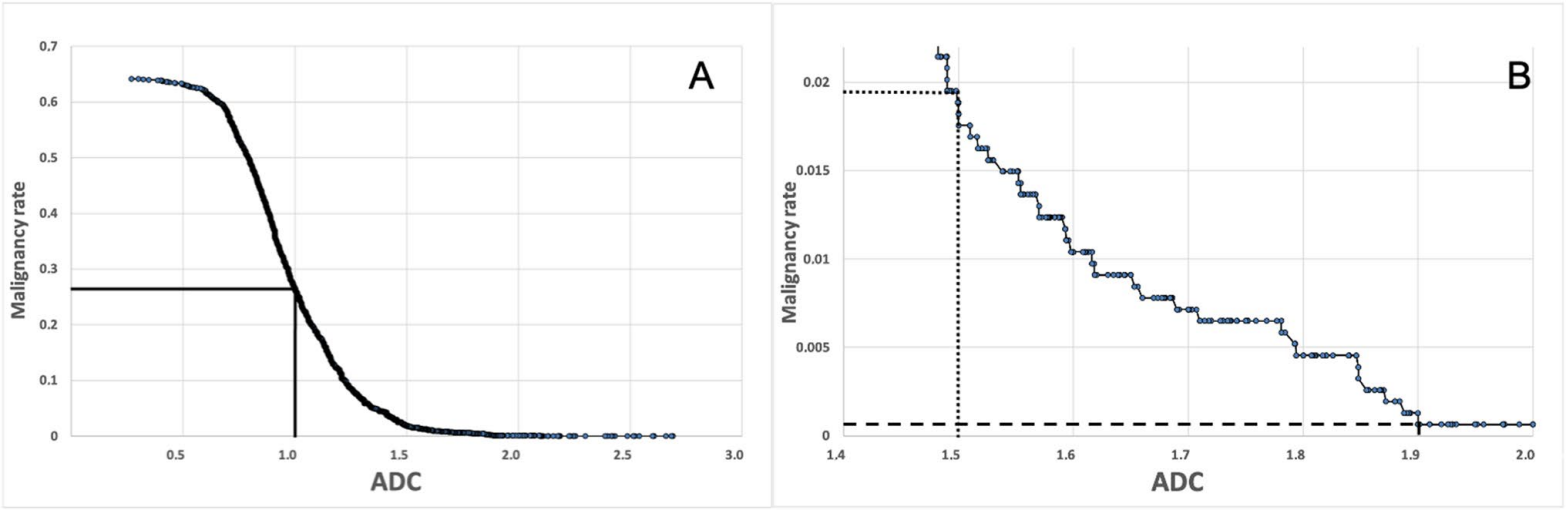
**A**, **B** Cumulative malignancy rates (*y*-axis) plotted against ADC values (*x*-axis). Dots on the curve represent each single case. Picture **B** depicts magnification of the malignancy rates below 0.025. The reference lines depict the ADC thresholds for the ADC categories: dashed line—1.9; dotted line—1.5; continuous line—1.0; ADC values are displayed in 10^−3^ mm^2^/s. Abbreviation: ADC—apparent diffusion coefficient

In a first step, the ADC values of each lesion were plotted against the cumulative malignancy rates in a simple curve (Fig. 2). In a second step, six basic ADC-B categories, based on these cumulative malignancy rates and in analogy to BI-RADS, were pre-defined. Thus, enhancing lesions were stratified according to cumulative malignancy thresholds established analogously to BI-RADS:

- Very high ADC (category ADC-B2, malignancy rate < 0.1%): As in BI-RADS 2, these lesions can be considered as benign with a very high diagnostic confidence and no further work-up would be needed.
- High ADC (ADC-B3, 0.1–2%): Comparable to BI-RADS 3, these lesions can be considered as probably benign. A short-term imaging follow-up should be suggested.
- Intermediate/low ADC (ADC-B4, 2–50%): As in BI-RADS 4/5, the probability of malignancy in this category is high enough to warrant a work-up with image-guided biopsy and histopathological analysis.
- Very low ADC (ADC-B5, > 50%).

In a third step, the ADC values at the defined cumulative malignancy threshold were drawn from the plotted curve (Fig. 2). In a fourth step, the cut-off value between categories 4 and 5 was adapted based on ROC curves. Here we aimed to distinguish between invasive and non-invasive breast cancers. A positive predictive value (PPV) of > 95% for invasive carcinomas was chosen as a fitting threshold. Since lesions in these two categories would be submitted to biopsy anyway, we deemed the possibility of distinguishing invasive from non-invasive carcinomas an interesting and probably useful tool. Finally, in a fifth step and in order to facilitate clinical application of the ADC thresholds, cut-off values were set to one decimal, thus leading to slightly different malignancy rates than previously defined in step two.

Category ADC-B0 applies to cases where ADC cannot be measured (e.g., due to artifacts), while category ADC-B1 applies to cases without an enhancing lesion on CE T1-weighted (analogous to BI-RADS 1). No such cases were included in the examined databases, and since there was no detectable lesion or measurable ADC, no ADC thresholds were applied for these categories.

### Statistical analysis

Statistical analysis was performed using SPSS 26.0 (IBM Corp.). With the exception of patient age, all calculations were performed on a per-lesion basis.

Means for the different lesion types were compared using one-way ANOVA and the Games-Howell post hoc test. Box plots were created to visualize the results. Benign and malignant lesions were also stratified by size (lesions ≤ 10 mm and > 10 mm), and means were compared between the size groups using the independent-samples *t* test. To test the robustness of ADC results within the heterogeneous database, a multivariable linear regression was performed using besides the final diagnosis (benign vs malignant) the center of ADC data origin, MRI unit field strength, and vendor and lesion size as covariates for the analysis.

Microsoft Excel (Microsoft Corp.) was used to plot the descending ADC values against the corresponding ascending cumulative malignancy rates to determine the thresholds of the ADC-B categories. In a further step, ROC was used to adapt the ADC cut-off between ADC-B categories 4 and 5.

The significance level was defined at 5%; thus, *p* values ≤ 0.05 were considered significant. No formal Bonferroni correction was applied as the number of statistical tests was limited and the number of cases high. Test results were interpreted considering clinical relevance of group differences to avoid overemphasis on spurious associations.

## Results

### Patient and lesion characteristics

Following the exclusion of 122 patients due to incomplete data (Fig. 1), 1625 female patients with 1736 lesions with a mean age of 55.9 years (SD 13.8) (Table 1) were included. A total of 1155 of 1736 lesions were malignant (66.5%) and 581/1736 lesions were benign (33.5%), 115/581 of which were high-risk lesions (19.8%). Of the malignant lesions, 1020/1155 (88.3%) were invasive breast carcinomas, 98/1155 (8.5%) were DCIS, 26/1155 (2.3%) were invasive mucinous carcinomas, and 11/1154 (0.9%) were other malignancies. The mean lesion size was 20.7 mm (SD 16, range 3–130 mm). Malignant lesions were significantly larger (mean 23.1 mm, SD 13 mm) than benign lesions (mean 15.9 mm, SD 17 mm, *p* < 0.001). There were 1333 (78.6%) mass and 362 (21.4%) non-mass lesions. No information about enhancement type was available for 41 lesions. Histopathological details are displayed in Table 3.

**Table 3:** Histopathological details of the included breast lesions

		*n*	%
Malignant subtypes		1155	66.5
	Invasive carcinoma—no special type	843	73.0
	Mucinous	26	2.3
	Papillary	7	.6
	Medullary	5	.4
	Cribriform	13	1.1
	Ductal carcinoma in situ	98	8.5
	Invasive lobular carcinoma	130	11.3
	Mixed invasive ductal/lobular carcinoma	22	1.9
	Other malignancy	11	1.0
High-risk subtypes		**115**	**6.6**
	Atypical ductal hyperplasia	4	3.5
	Lobular carcinoma in situ	18	15.7
	Columnar cell hyperplasia	4	3.5
	Radial scar/complex sclerosing adenosis	4	3.5
	Flat epithelial atypia	33	28.7
	Papilloma/papillomatosis	50	43.5
	Phyllodes	2	1.7
Benign subtypes		**466**	**26.8**
	Fibrosis/fibrocystic changes	180	38.6
	Adenosis/sclerosing adenosis	53	11.4
	Epithelial proliferation	17	3.6
	Fibroadenomatoid hyperplasia	14	3.0
	Fibroadenoma	132	28.3
	Fat necrosis/scar	7	1.5
	Apocrine metaplasia	3	.6
	Inflammation/mastitis	28	6.0
	Pseudoangiomatous stromal hyperplasia	6	1.3
	Other benign	26	5.6
Overall		**1736**	

Summaries of subgroups are given in bold

### ADC measurements

The mean ADC values were 1.45 × 10^−3^ mm^2^/s (SD 0.40, range 0.40–2.69) for the benign lesions, 1.37 × 10^−3^ mm^2^/s (SD 0.34, 0.60–2.43) for the high-risk lesions, and 0.95 × 10^−3^ mm^2^/s (SD 0.25, 0.27–2.10) for the malignant breast lesions. When separated by malignant subtypes, the mean ADC values were 0.92 × 10^−3^ mm^2^/s (SD 0.22, 0.27–1.90) for the invasive breast carcinomas (all types combined, with the exception of invasive mucinous carcinomas), 1.18 × 10^−3^ mm^2^/s (SD 0.30, 0.46–2.10) for the DCIS, 1.36 × 10^−3^ mm^2^/s (SD 0.30, 0.70–1.79) for the invasive mucinous breast carcinomas, and 0.91 × 10^−3^ mm^2^/s (SD 0.40, 0.39–1.85) for the other carcinomas.

The mean ADC differed significantly between benign and malignant lesions (*p* < 0.001), as well as between invasive breast carcinomas, DCIS, and benign lesions (*p* < 0.001). Mucinous breast carcinomas showed significantly higher ADC levels than other invasive carcinomas (*p* < 0.001), but not DCIS and benign lesions (*p* = 0.08–1.00). No significant difference could be found between the high-risk lesions and the benign lesions (*p* = 0.28).

When stratified by size, mean ADC values for the benign lesions showed minor but statistically significant differences between the subgroup of ≤ 10 mm (1.42 × 10^−3^ mm^2^/s) and > 10 mm (1.45, *p* = 0.007), while no significant difference could be found for the carcinomas (0.98 and 0.95, *p* = 0.28). Mean ADC values were significantly different between benign and malignant lesions within each size group (*p* < 0.001).

Multivariable linear regression revealed that only the final diagnosis (benign vs malignant) significantly contributed to ADC variation. A model incorporating the final diagnosis as covariate achieved an adjusted *R*-squared of 0.408 (explaining 40.8% of the ADC variation), while excluding the final diagnosis from the multivariable model led to an R-squared of 0.030 (explaining only 3% of the ADC variation) with lesion size as the only significant covariate.

### ADC categories

**Fig. 3:**
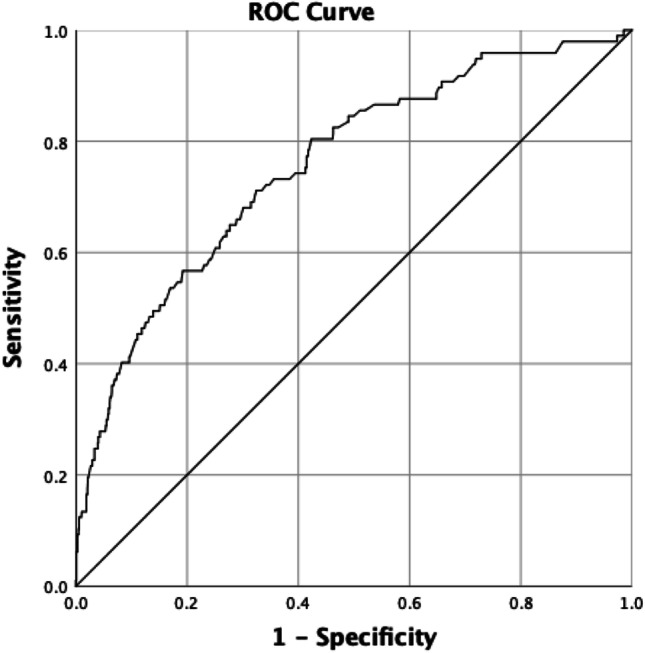
ROC curve comparing the ADC values of invasive and non-invasive breast cancers. This curve was used to determine the threshold between categories ADC-B4 and 5. The AUC of this curve is .757 (std. error .027, 95% CI .705–.809). The threshold was set at an ADC of 1.0 × 10^−3^ mm^2^/s, at which the PPV for invasive breast cancer was 95.8%; Abbreviations: ROC—receiver operating curve; ADC—apparent diffusion coefficient; AUC—area under the curve

The area under the ROC curve for invasive versus non-invasive carcinomas was 0.76 (std. error 0.027, 95% CI 0.73–0.78) (Fig. 3). The threshold between ADC-B4 and ADC-B5 was set at 1.0 × 10^−3^ mm^2^/s. At this threshold, the PPV for invasive breast carcinomas versus non-invasive DCIS was 95.8% (95% CI 0.94–0.97).

**Fig. 4:**
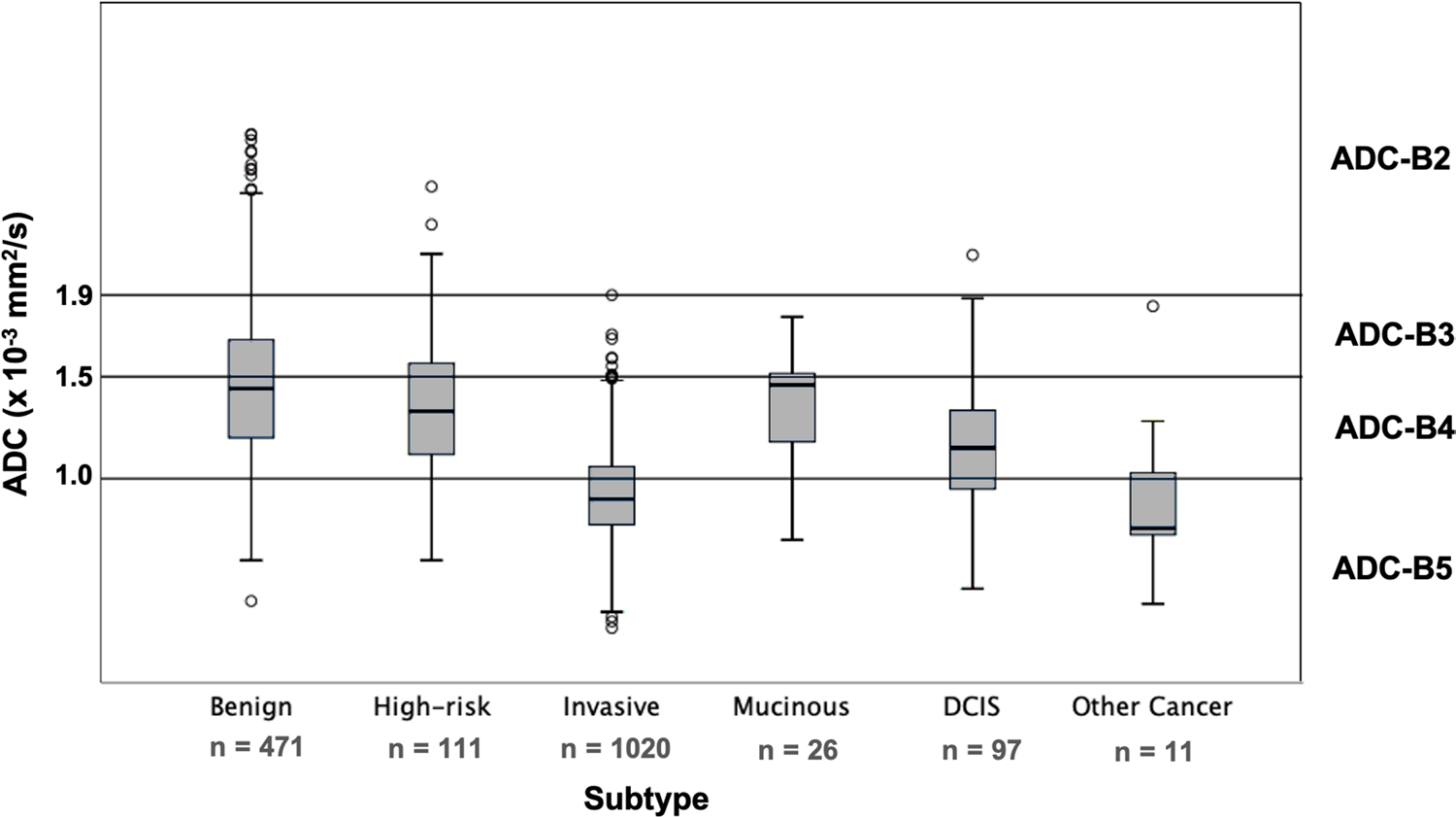
Boxplots displaying the ADC values of the different lesion subtypes in the corresponding ADC-B categories. The horizontal line within the box represents the median ADC, the box represents the IQR, and the whiskers represent 1.5 IQRs. ADC values are displayed in 10^−3^ mm^2^/s. Abbreviations: ADC—apparent diffusion coefficient; IQR—interquartile range; DCIS—ductal carcinoma in situ

**Fig. 5:**
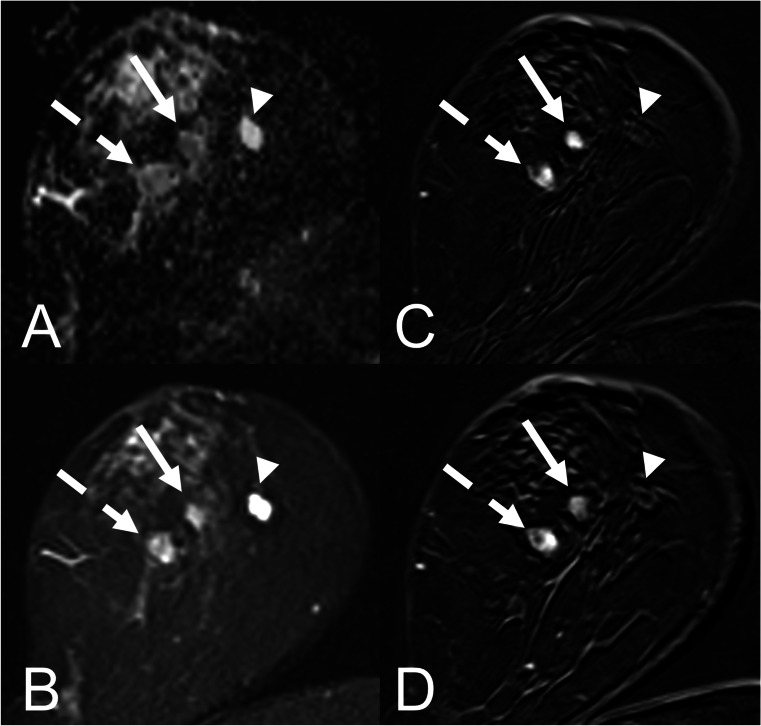
Three different lesion types at three different ADC-B categories in the right breast of a 59-year-old female patient who was admitted due to a BI-RADS III result in her screening mammography. **A** Axial ADC map (*b* = 0 and 800 s/mm^2^); **B** axial T2 STIR; **C** axial subtracted T1 VIBE 2 min after contrast administration; **D** axial subtracted T1 VIBE 7 min after contrast administration. Histopathology revealed the following lesion subtypes: adenosis (13 mm; dashed arrow; *ADC-B3*); fibroadenoma (10 mm; arrow; *ADC-B4*); and cyst (11 mm; arrowhead; *ADC-B2*). Abbreviations: ADC—apparent diffusion coefficient, STIR—short tau inversion recovery; VIBE—volumetric interpolated breath-hold examination

**Fig. 6:**
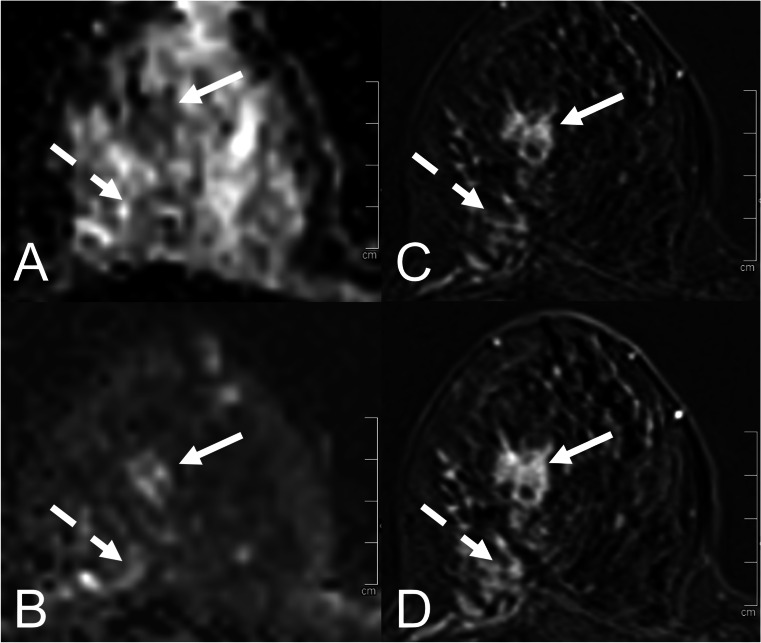
Two different lesion types at two different ADC-B categories in the right breast of a 68-year-old female patient who was admitted due to a suspicious lump in a physical examination and a BI-RADS 4 mammography result. **A** axial ADC map (*b* = 0 and 800 s/mm^2^); **B** axial diffusion-weighted image at a calculated *b* = 1400 s/mm^2^ (from *b* = 0 and 800 s/mm^2^; this is for the purpose of demonstration only, and images at the calculated *b* values were not used for the original study readings); **C** axial subtracted T1 VIBE 2 min after contrast administration; **D** axial subtracted T1 VIBE 7 min after contrast administration. Histopathology revealed the following lesion subtypes: DCIS (35 mm; dashed arrow; *ADC-B4*) and invasive lobular carcinoma (23 mm; arrow; *ADC-B5*). Abbreviations: ADC—apparent diffusion coefficient; VIBE—volumetric interpolated breath-hold examination; DCIS—ductal carcinoma in situ

Finally, the ADC thresholds for the ADC categories were ADC-B2: ADC ≥ 1.9 × 10^−3^ mm^2^/s, corresponding to a cumulative malignancy rate of < 0.1%; ADC-B3 1.5 to < 1.9 × 10^−3^ mm^2^/s, malignancy rate 0.1–1.7%; ADC-B4: 1.0 to < 1.5 × 10^−3^ mm^2^/s, malignancy rate 1.7–24.5%; and ADC-B5: < 1.0 × 10^−3^ mm^2^/s, malignancy rate > 24.5%. (Table 4, Fig. 4). Image examples for different ADC-B categories are presented in Figs. 5 and 6.

**Table 4:** ADC-B categories as suggested by the results of this analysis, with according cumulative malignancy rates, ADC ranges, and numbers of benign and malignant cases in each category. ADC values are displayed in 10 − 3 mm^2^/s

ADC-B category	Cumulative malignancy rate	ADC range	Benign (%)	Malignant (%)	*n*
0 (ADC not measurable)	–	–	–	–	0
1 (no enhancing lesion)	–	–	–	–	0
*2 (very high)*	< 0.1%	≥ 1.9	67 (97.1)	2 (2.9)	69
*3 (high)*	0.1–1.7%	1.5 to < 1.9	159 (85.0)	28 (15.0)	187
*4 (intermediate/low)*	1.8–24.5%	1.0 to < 1.5	288 (42.2)	395 (57.8)	683
*5 (very low)*	> 24.5%	< 1.0	67 (8.4)	730 (91.6)	797

ADC values are displayed in 10^−3^ mm^2^/s

*ADC* apparent diffusion coefficient

## Discussion

The apparent diffusion coefficient is a valuable marker for the diagnosis of breast lesions. Using a large multicenter database containing individual patient and lesion data obtained with different hardware and scanning parameters, we developed an easy-to-use breast apparent diffusion coefficient (ADC-B) categorization system to complement the American College of Radiology Breast Imaging Reporting and Data System (BI-RADS) in the assessment, documentation, and reporting of ADC values in contrast-enhancing breast lesions on MRI, which could seamlessly be integrated into MRI BI-RADS reporting. The ADC-B categorization includes a rule-out malignancy category at 99.9% in category ADC-B2 and differentiates invasive from non-invasive breast carcinoma with a PPV of 95.8% between categories ADC-B4 and 5.

Despite the well-researched capabilities of ADC in breast imaging [1–5], its implementation into clinical routine is still work in progress: a lack of standardization and a broad range of reported ADC values and thresholds for breast lesion subtypes have hindered standardized and comparable reporting of ADC values in clinical practice. Also considering inter-reader variation, a single ADC threshold may be too simplistic for use in clinical practice. As with all biomarkers, there is a gradual increase of the probability of malignancy with increasing ADC values. Therefore, an international working group has recently suggested dividing the ADC into categories [7] in order to make reporting more practicable. While the working group has suggested ADC categories based on the results of a meta-analysis, we collected original patient and lesion data from multiple study samples from different centers, with different hardware, DWI acquisition parameters, and patient collectives to create an ADC-B category system that can be applied to all of the researched populations.

As previously reported [2], there was no feasible ADC threshold to exclude malignancy with a certainty of 100%, since particular carcinoma subtypes, such as invasive mucinous carcinomas [12] or DCIS [13], may present with ADC values overlapping with those of benign lesions. Thus, we deemed a malignancy rate of < 0.1% reasonable for the rule-out category ADC-B2.

Lesions in category ADC-B3 come with a cumulative malignancy rate < 1.7%. This threshold was determined by finding an ADC threshold for a cumulative malignancy rate of 2% and rounding this threshold to one decimal, since a threshold with more than one decimal is not feasible in clinical practice: While inter-reader variability is generally low for ADC measurements in the breast, a level of agreement up to the second decimal is probably unreachable [14].

The threshold between the categories ADC-B4 and 5 was determined by calculating an ROC curve, in order to distinguish between invasive breast carcinomas and non-invasive DCIS with a PPV of 95%. DCIS is a common [15] non-invasive breast cancer type with a small chance of becoming clinically significant, and usually presents with higher ADC values than invasive carcinomas [4]. Since there are ongoing clinical trials on whether DCIS should be treated differently than invasive breast carcinomas [16–19], the possibility of differentiating these entities would be of clinical relevance. While category ADC-B4 comes with a relatively cumulative malignancy rate of 24.5%, possibly leading to unnecessary biopsies, this rate lies within those of the BI-RADS category 4 (which lies between 2 and 95%), for which biopsy is suggested anyway. And despite the seemingly low cumulative malignancy rate at its upper threshold, the prevalence of malignancy in lesions within category ADC-B5 is 91.6%.

In addition, we suggest categories that cover for cases without enhancing lesions (ADC-B1) and cases in which the ADC cannot be evaluated, e.g., due to artifacts (ADC-B0). These categories could prove particularly helpful for audit purposes.

While the ADC-B categories are derived from multicenter individual lesion and patient data and are, therefore, as a lowest common denominator, applicable to all of the included subpopulations, it has to be noted that these thresholds are not set in stone: with the addition of more ADC data from other sources, they may well be adapted in the future. This could especially be the case if further standardization of DWI, as suggested by the EUSOBI DWI working group [7], for example, prevails. Our multivariable analysis of our heterogeneous database, however, did not reveal a significant influence of technical acquisition differences on ADC variability and therefore supports the robustness of the results presented here. Additionally, refined categories could be developed for special indications such as breast cancer screening.

The ADC values found in this study are comparable to those previously reported. Significantly higher values were found in benign than in malignant lesions [2, 4], with the exception of invasive mucinous carcinomas. This can be attributed to the low cellularity and mucine content of this entity [12, 20, 21]. Still, mucinous carcinomas were predominantly found in category ADC-B4 (Fig. 4), and thus require biopsy.

No significant ADC differences could be found between benign lesions with and without high-risk criteria (uncertain malignant potential). The microstructural changes in benign high-risk lesions do not seem to have an objective influence on the observed ADC. In contradiction, Parsian et al reported significant differences between high-risk lesions and other benign subtypes [22]. However, in Parsian’s study, > 80% of the high-risk lesions were atypical ductal hyperplasias, while the most common subtype in this study was papillomas/papillomatosis (43.5% of the high-risk lesions). Thus, it can be concluded that while some high-risk subtypes may present with lower ADC values than benign breast lesions, this cannot be generalized.

Furthermore, while ADC is the most commonly used quantification method for DWI, it is a very simple and rather crude approximation of water diffusion properties in tissue. There are newer techniques such as intravoxel incoherent motion or non-Gaussian diffusion models that should better represent this diffusion and show comparable diagnostic performance [1]. While these methods might someday outperform ADC, they have not yet found their way into routine clinical practice. Comparable categorization systems could also be developed for parameters derived from these advanced diffusion models in the future.

This study has some limitations: firstly, the heterogeneity of the underlying data. While our multivariable analysis shows that only diagnosis (benign vs malignant) was a relevant factor influencing ADC values and thus ADC values were robust given the equipment and methods employed in this study, we do not provide an in-depth analysis of ADC confounders. Though this was outside the scope of this study, dedicated analyses, e.g., on the relevance of standardizing diffusion times, are warranted. From a clinical practice point of view, we see the inhomogeneity of the included patient samples and acquisition techniques as a strength, since this inhomogeneity represents the clinical reality and the established ADC thresholds can therefore be used in different clinical settings. This should not imply that standardization is not required but rather that the proposed ADC-B classification is already applicable. Secondly, the examined study samples included only lesions that have been biopsied for a definite diagnosis. Since lesions categorized as BI-RADS 2 or 3 are usually not biopsied, this may have led to a potential bias of lower malignancy rates in high ADC categories due to false-positive low ADC. However, since there is no rule-in criterion for malignancy anyway, this should not lower the applicability of our results. Another point of interest may be the stratification of ADC-B by lesion appearance as mass or non-mass. The aim of this study was to provide a simple ADC categorization system including a rule-out category applicable to mass and non-mass lesions alike. Further independent validation studies may show whether a more sophisticated approach provides additional value despite complicating application in clinical practice. Thirdly, we did not test the combination of the proposed ADC categories in combination with conventional (enhanced or unenhanced) breast MRI, since we felt that this exceeds the scope of this study.

In conclusion, the breast apparent diffusion coefficient (ADC-B) categorization system provides a simple and widely applicable categorization scheme to complement MRI BI-RADS criteria for assessment, documentation, and reporting of ADC values in contrast-enhancing breast lesions on MR imaging.

## Supplementary Information

Below is the link to the electronic supplementary material.

Supplementary file1 (PDF 84 KB)

## Abbreviations

ADC-B: Breast apparent diffusion coefficient
DCIS: Ductal carcinoma in situ
EUSOBI: European Society of Breast Imaging

## Declarations

### Guarantor

The scientific guarantor of this publication is Pascal Baltzer.

### Statistics and biometry

One of the authors (Pascal Baltzer) has significant statistical expertise.

### Study subjects or cohorts overlap

Since this is a retrospective analysis of multiple study populations, some of the original data analyzed in this study have, at least in part, already been analyzed and published with different research questions.

The according most recent publications are referenced below:

-Bickel, H. et al Quantitative apparent diffusion coefficient as a noninvasive imaging biomarker for the differentiation of invasive breast cancer and ductal carcinoma in situ. *Investigative Radiology* 50, 95–100, https://doi.org/10.1097/rli.0000000000000104 (2015).

-Clauser, P. et al Is there a systematic bias of apparent diffusion coefficient (ADC) measurements of the breast if measured on different workstations? An inter- and intra-reader agreement study. Eur Radiol, https://doi.org/10.1007/s00330-015-4051-2 (2015).

-Dietzel, M. et al A multicentric comparison of apparent diffusion coefficient mapping and the Kaiser score in the assessment of breast lesions. *Investigative Radiology* 56, 274-282, https://doi.org/10.1097/rli.0000000000000739 (2021).

-Spick, C., Pinker-Domenig, K., Rudas, M., Helbich, T. H. & Baltzer, P. A. MRI-only lesions: application of diffusion-weighted imaging obviates unnecessary MR-guided breast biopsies. Eur Radiol 24, 1204-1210, https://doi.org/10.1007/s00330-014-3153-6 (2014).

-Clauser, P. et al Diffusion-weighted imaging allows for downgrading MR BI-RADS 4 lesions in contrast-enhanced MRI of the breast to avoid unnecessary biopsy. *Clinical Cancer Research*, 1941-1948, https://doi.org/10.1158/1078-0432.Ccr-20-3037 (2021).

-Marino, M. A. et al A simple scoring system for breast MRI interpretation: does it compensate for reader experience? Eur Radiol 26, 2529-2537, https://doi.org/10.1007/s00330-015-4075-7 (2016)

-Molinari, C. et al MR mammography using diffusion-weighted imaging in evaluating breast cancer: a correlation with proliferation index. Radiol Med 120, 911-918, https://doi.org/10.1007/s11547-015-0527-z (2015).

-Baltzer, A., Dietzel, M., Kaiser, C. G. & Baltzer, P. A. Combined reading of contrast enhanced and diffusion weighted magnetic resonance imaging by using a simple sum score. *European Radiology* 26, 884-891, https://doi.org/10.1007/s00330-015-3886-x (2016).

-Baltzer, P. A. T. et al Diffusion-weighted imaging (DWI) in MR mammography (MRM): clinical comparison of echo planar imaging (EPI) and half-Fourier single-shot turbo spin echo (HASTE) diffusion techniques. Eur Radiol 19, 1612-1620 (2009).

-Dietzel, M. et al Breast MRI in the era of diffusion weighted imaging: do we still need signal-intensity time curves? Eur Radiol 30, 47-56, https://doi.org/10.1007/s00330-019-06346-x (2020).

-Moschetta, M., Telegrafo, M., Rella, L., Stabile Ianora, A. A. & Angelelli, G. Effect of gadolinium injection on diffusion-weighted imaging with background body signal suppression (DWIBS) imaging of breast lesions. Magn Reson Imaging 32, 1242-1246, https://doi.org/10.1016/j.mri.2014.07.014 (2014).

As opposed to these previous publications, we conjugated the original data from the different imaging centers to develop an easily and generally applicable ADC categorization system (bADC).

### Methodology

• retrospective

• cross sectional

• multicenter study
