# Supplementary material for: Introduction of a breast apparent diffusion coefficient category system (ADC-B) derived from a large multicenter MRI database
Source: Eur Radiol. 2023 May 11;33(8):5400–10. doi: 10.1007/s00330-023-09675-0 (PMC10326122; doi:10.1007/s00330-023-09675-0)
Supplement: Supplementary file 1 — Supplementary file1 (PDF 84 KB) [file 330_2023_9675_MOESM1_ESM.pdf]

ELECTRONIC SUPPLEMENTAL MATERIAL

Introduction of a Breast Apparent Diffusion Coefficient Category System (ADC-B) Derived from a Large Multicenter MRI Database

Supplemental Table 1: Previously publishes studies on the patients included in this study.

Study sample	Years of acquisition	Year of first publication	n readers	Reader experience	Quality control	Reference no	Comment
C1-P1	2007 - 2011	2015	1 + 1	3 + 6 years	Consensus	*4*	
C1-P2	2007 - 2012	2014	1	11 years	-	*24*	
C2	2013 - 2014	2016	1	13 years	-	*26*	*DWI data unpublished*
C3	2013	2015	2	5 years	Consensus	*14*	
C4-P1	2007 – 2008	2009	2	5 + 6 years	Consensus	*29*	
C4-P2	2007 - 2009	2016	2	12 + 13 years	Consensus	*28*	
C4-P3	2007	2020	1	16 years	-	*30*	
C5-P1	2017 – 2019	unpublished	2	10 + 25 years	Consensus	unpublished	
C5-P2	2017 – 2019	unpublished	2	10 + 25 years	Consensus	unpublished	
C6	2011 - 2013	2014	2	5 years	Consensus	*31*	
C7	2016 - 2017	unpublished	3	5 - 15 years	-	unpublished	
